# The chemokine CXCL13 is a key regulator of B cell recruitment to the cerebrospinal fluid in acute Lyme neuroborreliosis

**DOI:** 10.1186/1742-2094-6-42

**Published:** 2009-12-30

**Authors:** Tobias A Rupprecht, Andreas Plate, Michaela Adam, Manfed Wick, Stefan Kastenbauer, Caroline Schmidt, Matthias Klein, Hans-Walter Pfister, Uwe Koedel

**Affiliations:** 1 Department of Neurology, Ludwig-Maximilians University, Marchioninistr. 15, 81377 Munich, Germany; 2 Department of Clinical Chemistry, Ludwig-Maximilians University, Marchioninistr. 15, 81377 Munich, Germany

## Abstract

**Background:**

The chemokine CXCL13 is known to dictate homing and motility of B cells in lymphoid tissue and has been implicated in the formation of ectopic lymphoid tissue in chronic inflammation. Whether it influences B cell trafficking during acute infection, is largely unclear. In previous studies, we showed that (I) CXCL13 levels are markedly increased in the B cell-rich cerebrospinal fluid (CSF) of patients with acute Lyme neuroborreliosis (LNB), and (II) CXCL13 is released by monocytes upon recognition of borrelial outer surface proteins by Toll-like receptor 2. Here, we assessed the role of CXCL13 - in comparison to other chemokines - in the recruitment of B cells to the CSF of patients with acute LNB.

**Methods:**

Measurement of chemokines was done by ELISA. B cells were isolated from whole blood using magnetic cell separation (MACS). For migration experiments, a modified Boyden chamber assay was used and the migrated B cells were further analysed by FACS. The migration was inhibited either by preincubation of the CSF samples with neutralizing antibodies, heating to 60°C, removal of proteins >3 kDa, or by pre-treatment of the B cells with pertussis toxin. The principal statistical tests used were one-way analysis of variance and Bonferroni test (chemokine measurements) as well as paired Student's t-test (migration experiments).

**Results:**

Measurements of chemokine levels revealed an increase in three of the four known major B cell chemoattractants CXCL13, CCL19 and CXCL12 in LNB CSF. The CXCL13 CSF:serum ratio, as a measure of the chemotactic gradient, was substantially higher than that of CCL19 and CXCL12. Moreover, the chemotactic activity of LNB CSF was reduced up to 56% after preincubation with a neutralizing CXCL13 antibody, while combined preincubation with antibodies against CXCL13, CCL19, and CXCL12 did not lead to further reduction. Since treatment with pertussis toxin, heating to 60°C, and removal of proteins >3 kDa abrogated the chemotactic activity, further not yet identified chemokines seem to be involved in B cell recruitment to LNB CSF.

**Conclusion:**

Combined, our study suggests a key role of CXCL13 in B cell migration to sites of infection as shown here for the CSF of LNB patients.

## Background

The field of function of CXCL13 has been constantly growing since its discovery in 1998 [1]. Initially, the essential role of CXCL13 was seen in the establishment and maintenance of lymphoid tissue microarchitecture [2]. Accordingly, CXCL13 deficient mice fail to develop lymph nodes [3], and B-cell homing to lymph node follicles requires CXCL13 and its exclusive receptor CXCR5 [4]. Some years later, evidence for a role in the formation of ectopic lymphoid tissue in chronic inflammation such as multiple sclerosis or rheumatoid arthritis was also found [5,6]. Finally, the detection of CXCL13 expression in *Helicobacter pylori *gastritis [7], pulmonary tuberculosis [8] or *Bartonella henselae *infection [9] suggested a role of this chemokine in chronic bacterial infections as well. Its influence on leucocyte migration to the site of infection, however, has not been evaluated so far.

Recently, we and others observed a strong up-regulation of CXCL13 expression in an acute bacterial infection, in Lyme neuroborreliosis (LNB) [10,11]. In LNB, the spirochete *Borrelia burgdorferi *(*B.b*.) invades the cerebrospinal fluid (CSF) [12]. The host immune system reacts to the invading spirochetes with a local inflammation, leading to an intrathecal accumulation of leucocytes. A hallmark of this CSF-pleocytosis in LNB is the accumulation of activated B cells and plasma cells. The percentage of B cells in the CSF of LNB patients reaches up to 80%, clearly exceeding other CNS infections [13]. B cells show a substantial migration only to very few chemokines, namely, CCL19, CCL21, CXCL12, and CXCL13 [14].

In previous studies, we measured high concentrations of CXCL13 in the CSF of patients with LNB, even before the intrathecal production of *B.b*.-specific antibodies has started [10,15]. Cell culture experiments have shown, that PBMC produce CXCL13 in response to an incubation with *B.b*. through the interaction of the TLR2 receptor of the innate immune system with spirochete outer surface proteins [16]. This *in vitro *study is further supported by findings in the rhesus monkey model of LNB, where the CXCL13 expression at the spinal nerve roots correlated with the spirochete load and resident immune cells have been identified as source of this chemokine [11,17]. Based on these data, we speculated that *in vivo*, the high intrathecal concentration of CXCL13 in LNB patients directs the B cells to the CSF, leading to the observed B cell enriched CSF pleocytosis [12].

To further assess the role of CXCL13 for the B cell rich infiltrate in the CSF of LNB patients, we (1) examined the chemotactic activity of CSF samples from patients with LNB on human B cells, compared with that of patients with a non-inflammatory CNS diseases (NIND), and neurosyphilis (NS) as another spirochete CNS disease in a chemotaxis assay, (2) determined the concentrations of CXCL13 and other B cell attracting chemokines in CSF/serum pairs of the three patient groups, and (3) tried to elucidate the contribution of these chemokines to the chemotactic activity of LNB by using specific neutralizing antibodies.

## Methods

### B-cell isolation and stimulation

Human peripheral blood mononuclear cells were extracted from venous blood samples from a healthy male donor using ficoll gradient centrifugation. Subsequent untouched B cells were isolated by Midi Macs System^® ^(Miltenyi Biotec, Bergisch-Gladbach, Germany). The purity of the (CD19^+^) B cells, examined repetitively before and during the experiments by a FACS analysis, was always higher than 98.4%. More than 95% of isolated cells were shown by the Trypan blue exclusion test to be viable. In order to stimulate the B cells to increase their migratory ability, they were incubated for 16 hours at 37°C and 5% CO_2 _in RPMI1640 medium supplemented with 10%FCS, 100 units penicillin and 100 μg streptomycin/ml, 20 ng/ml IL4 and 1 μg/ml CD40L according to [14]. Thereafter, the cells were pelleted again and diluted to a working concentration of 2.6 × 10^6 cells/ml for the migration assays and 2.1 × 10^6 cells/ml for the inhibition assays.

### CSF/blood samples

Blood was drawn and lumbar puncture was performed for diagnostic purposes after the patient's informed consent was obtained. All samples were frozen at -30°C. Paired CSF and blood samples were obtained from the following groups:

(i) 14 patients (9 males) with non-inflammatory CNS disease (NIND). The mean cell count was 1.8 [0.3-4.7] cells/μl, and the mean CSF/serum albumin ratio was 8.7 [3.3-31.0]. The mean age was 64.7 [48-74] years. Three of these patients suffered from chronic pain, two others had an epileptic seizure, and the further patients were diagnosed amyotrophic lateral sclerosis, vascular compression of the brain stem, Parkinson's disease, transverse myelitis, Guillain-Barré syndrome, multiple sclerosis, herniated vertebral disc, somatoform disorder or progressive ataxia.

(ii) 18 patients (11 males) with acute (duration of symptoms < 6 months) Lyme neuroborreliosis (LNB) before initiation of therapy. The mean cell count was 146 [1-600] cells/μl (for details, please refer to table 1), the mean CSF/serum albumin ratio was 26.0 [3.9-43.9]. The mean age was 57.8 [15-72] years. The diagnosis of LNB was based on the following criteria: 1) typical clinical picture (e.g. meningoradiculitis, cranial neuritis or meningitis) 2) lymphocytic CSF pleocytosis, and 3) increased intrathecal *B.b*.-specific antibody production. Detailed data concerning basic CSF findings as well as the *B.b*.-specific antibody index and the percentage of plasma cells are given in table 1.

**Table 1:** Clinical data and basic CSF parameters of the LNB patients

**Pat. Nr**.	Gender f/m	Age [y]	Clinical syndrome	Cell count [/μl]	Plasma cells [%]	**Albumin quotient**^**1**^	**Antibody index**^**2**^
**1**	f	69	BS	53	15	43.9	1.7

**2**	m	15	CNP, M	600	0	5.4	4

**3**	f	70	BS	90	0	4.83	n.d.^+^

**4**	m	62	BS, CNP	246	16	39.7	7.3

**5**	f	69	BS	109	5	15.8	13.3

**6**	f	61	BS	217	1	37.9	40.7

**7**	m	66	BS, CNP	16	0	8.9	<1.5^#^

**8**	m	67	BS, M	52	20	10.3	34

**9**	m	61	BS	1*	8	30.3	10.9

**10**	m	72	CNP	166	3	21.6	62.7

**11**	m	59	BS	100	0	23.4	2.8

**12**	m	38	M	117	11	26	168

**13**	m	55	BS, CNP	368	n.d.	19.1	38.6

**14**	m	50	CNP	12	0	4.5	7.7

**15**	f	46	BS	14	0	3.9	1.8

**16**	m	59	BS	30	0	13.2	2.5

**17**	f	59	BS	255	5	33.2	17.8

**18**	f	62	CNP, M	180	1	10.5	4.3

BS: Bannwarth's syndrome (meningopolyradiculitis), CNP: Cerebral nerve palsy (Cranial neuritis), M: Meningitis. ^1^CSF/serum albumin quotient, ^2^*B.b*.-specific antibody index. * = cell count at follow up lumbar puncture (8 days later) showed 70 leucocytes/μl and an increasing antibody index (27.5). ^+ ^antibody index initially not done, but on repeated lumbar puncture 13 days later, the CSF showed an *B.b*.-specific antibody index of 9.34 (and cell count of 102/μl). ^# ^antibody index at follow up 4 days later 2.1, and 12 days later 4.8 (and cell count of 13, and 17, respectively).

(iii) 14 patients (13 males) with neurosyphilis (NS). The mean cell count was 17.4 [5-45] cells/μl and the mean CSF/serum albumin ratio was 5.9 [3.2-12.5]. The mean age was 31.8 [19-48] years. The diagnosis was based on serological proof of a syphilitic infection (positive *Treponema *particle agglutination and fluorescent treponemal antibody absorbed tests) and an elevated CSF white blood cell count according to the criteria described by Marra et al. [18]. All patients with syphilis were in the second stage of disease (secondary syphilis) and negative for the human immunodeficiency virus (HIV).

### Migration assay

The migration assay was performed in a disposable 96-well system (ChemoTx, Neuroprobe) with polycarbonate membrane filters (5 μm pore). 30 μl of the CSF samples were added to the lower chamber and 50 μl B cell solution was added onto the membrane. The chamber was incubated for 1 h at 37° in humidified air with 5% CO_2_. After incubation, the migrated cells in the lower chamber were counted in a Fuchs-Rosenthal-chamber.

For better comparison of the results from different experiments, a migration index was calculated. We used medium as negative control value and 500 ng/ml rhCXCL13 (R&D Systems, Minneapolis, MN, USA) as a positive control.

The results are presented in per cent. A MI of 100% implicates, that the sample had the same chemotactic effect as 500 ng rhCXCL13.

### FACS analysis

Anti-human monoclonal antibodies against CD19 (PC5, J3-119, IgG1) and CD27 (PE, 1A4, IgG1), obtained by Immunotech (Marseille Cedex, France) were used. For flow cytometry 50 μl cell suspension (2 × 10^6^/ml) of isolated B cells and either B cells, which did migrate to rhCXCL13 or LNB CSF, and those that have not migrated were incubated with 5 μl of CD19 and 10 μl of CD27 each for 15 minutes at room temperature in the dark. 1 ml PeliLyse lysing reagent (Hiss Diagnostics GmbH, Freiburg, Germany) was added to each of the tubes and incubated for another 10 minutes in the dark at room temperature. The cells were washed and resuspended in 200 μl PBS (Merck KGaA, Darmstadt, Germany) and afterwards immediately analysed.

Analysis was performed on a FACS Canto II flow cytometer (Becton Dickinson, San Jose, USA) using FACSDiva software. 10,000 events were acquired. Data were displayed as two colour plots (CD19/CD27). The data of migrated B cells were compared with those of non migrated B cells.

### ELISA

From the CSF/serum samples used for the migration assays, sufficient volume for the quantitative measurement of the chemokines CXCL12, CCL19 and CCL21 was available from 12 NIND (mean cell count 1.89/μl, mean total CSF protein 553 mg/l, mean age 65.2 years, and 58% male), 14 NB (mean cell count 375.6/μl, CSF protein 1,186 mg/l, mean age 56.4 years, and 64% male) and all NS patients. CXCL13 levels in CSF and serum samples were measured in all samples used in the migration assays. A part of the samples have already been analysed for CXCL13 before [10,16]. The measurements were done by ELISA (R&D) according to the recommendations of the manufacturer.

### Inhibition assay

To investigate the chemotactic impact of CXCL13, CXCL12 and CCL19 on the human B cells, the respective chemokines in the CSF samples from LNB patients were blocked with neutralizing antibodies. For the isolated blockade of CXCL13 activity, 10 μg/ml of the neutralizing polyclonal CXCL13 antibody or an isotype control antibody (10 μg/ml) (all R&D) was used. For triple inhibition assays, we added additional 10 μg/ml polyclonal CCL19 and 50 μg/ml polyclonal CXCL12 antibody or analogous amounts of isotype control antibodies. In pre-tests, we could show that the CXCL13 and CCL19 antibodies inhibit >99% of the chemotactic effect of 50 ng/ml rhCXCL13 and rhCCL19, respectively. In contrast, though testing several available neutralizing antibodies in different concentrations, the blockade of CXCL12 activity only reached 42%. Cross reactivity between all applied antibodies was tested and not observed. All samples were preincubated on a shaker with the respective antibody for 30 min at RT. As negative control, we used a mixture from 12 CSF samples from NIND patients as they would represent the baseline of migratory activity in vivo, 500 ng/ml rhCXCL13 served as positive control. To perform the assay, sufficient CSF volume from 12 patients was available, but only 9 samples were tested as their MI was higher than all NIND samples. In addition, 3 LNB CSF samples were either heated at 60°C for 10 minutes to denature protein structures, filtrated for 90 min at 14 g using a membrane with a 3 kDa Nominal Molecular Weight Limit (Millipore), or the B cells were preincubated for 90 min with 5 μg/ml PTx. All experiments were performed in duplicate.

### Statistics

In order to assess statistical significant differences of the chemotactic activity of CSF samples obtained from patients with NIND, LNB, or NS on isolated B cells we used one way analysis of variance (ANOVA) and Bonferroni adjustment. This statistical test was also used to measure the significant differences between the chemokine concentrations of these groups in the ELISA assays. The paired t-test was used to measure the differences in the migration behaviour of B cells between antibody- and isobody-control groups in the inhibition assays. The data were expressed as mean ± SD.

## Results

### Chemotactic activity of CXCL13 and CSF from LNB patients on B cells

**Figure 1:**
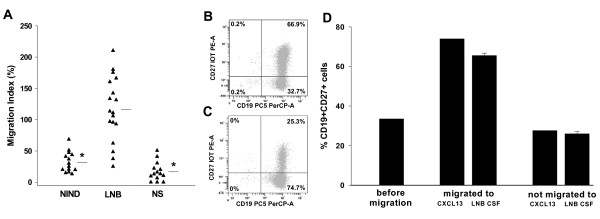
**Migration of B cells to CSF samples**. (A) migration index of CD19^+ ^B cells to CSF samples from different patient groups, horizontal bars represent the mean value, *: p < 0.001. (B) and (C) exemplary result of a FACS analysis of (B) migrated or (C) not-migrated CD19^+^CD27^+ ^B cells towards the CSF sample of a LNB patient. (D) percentage of migrated vs. not-migrated CD27^+^CD19^+ ^B cells, using either CSF-samples of patients with LNB (n = 3) or 500 ng/ml rhCXCL13 (mean of two independent experiments, done in duplicate) as chemotactic agents.

First, we determined the chemotactic impact of recombinant human (rh)CXCL13 on human B cells in a well established chemotaxis assay, the modified boyden chamber [19,20]. rhCXCL13 had a dose-dependent chemotactic activity by increasing the amount of migrating B cells between 1.5-fold (at 5 ng/ml) and 7.4-fold (at 500 ng/ml) in comparison to the medium control (data not shown). The proportion of mature, CD27+ B cells was 73.9% in the migrated cells, while only 25.5% of the non-migrated cells were expressing this activation marker (Fig. 1d).

Next, we analysed the migratory activity of CSF samples from different patient groups (NIND, LNB, and NS) on B cells. The migratory index (MI, for details please refer to the Methods section), was clearly higher for the LNB group (MI = 116) than for both other patient groups (32.1 for NIND and 16.8 for NS, p < 0.001) (Fig. 1a). Similar to rhCXCL13, 65.5% of the B cells that have migrated towards the CSF of LNB patients were expressing CD27, in contrast to 26% of the non-migrating cells (Fig. 1b-d). Together, these findings indicate, that predominantly mature, activated B cells are attracted by both, rhCXCL13 (in concentrations as found in the CSF of LNB patients) and the CSF of LNB patients.

### Concentration of B cell-attracting chemokines in CSF/serum sample-pairs

**Figure 2:**
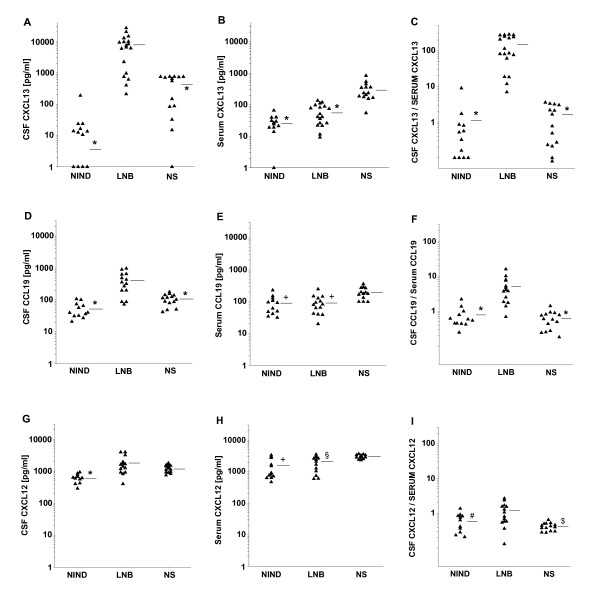
**Concentration of Chemokines in CSF samples**. Concentration of the respective chemokines in either CSF- (A, D, and G) or serum-samples (B, E, and H) of the different patient groups, and accordingly the calculated CSF-to-serum ratio (C, F, and I), horizontal bars represent the mean value, *: p < 0.001, $: p = 0.003, and #: p = 0.021 vs. LNB, and +: p < 0.001, and § p = 0.013 vs. NS

To identify the immune factors responsible for the increased chemotactic activity of CSF samples from LNB patients, we measured the concentration of the four major B cell attracting chemokines CXCL13, CCL19, CXCL12 and CCL21 in CSF samples from all three patient groups (LNB, NIND and NS). The concentration of CXCL13 was significantly increased in the CSF of LNB patients (mean = 7,864 pg/ml, range 209-26,769) compared to NIND (25 pg/ml, 0-188) and NS patients (426 pg/ml, 0-750, Fig. 2a). A significant higher level in LNB patients than in both other patient groups was also found for CCL19 (400 pg/ml, 72-959 vs. 52 pg/ml, 21-107 in NIND and 104 pg/ml, 42-176 in NS patients, Fig. 2d). For CXCL12, the difference was significant between LNB (1,797 pg/ml, 404-3,963) and NIND (564 pg/ml, 0-938), but not NS (1,239 pg/ml, 759-1,818, Fig. 2g). For CCL21 (1 pg/ml, 0-13 in LNB vs. 17 pg/ml, 0-140 in NIND and 12 pg/ml, 0-75 in NS) no significant difference between the patient groups was observed. These findings argue for a role of CXCL13, CCL19, and CXCL12, but not CCL21 for B cell recruitment in LNB.

**Figure 3:**
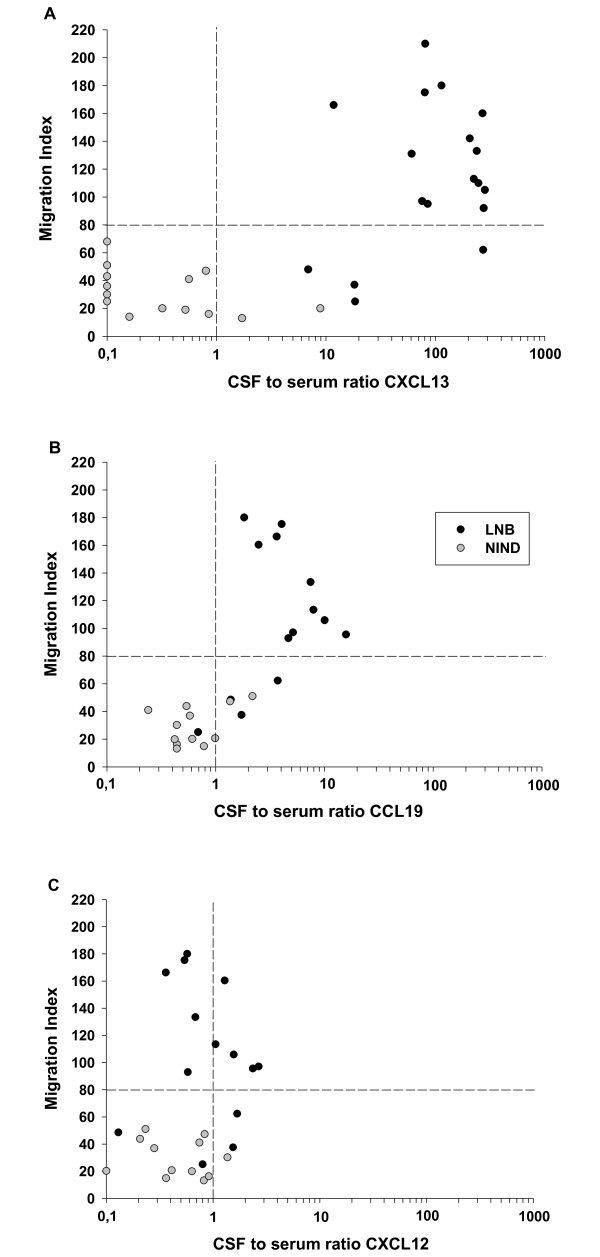
**Correlation of the CSF-to-serum ratio of chemokines with the migration index**. Correlation of the CSR of the different chemokines with the MI of the respective CSF sample as measured in the migration assay. A cut-off for the MI was set at 80, as all CSF samples from NIND patients (and accordingly no inflammatory cell infiltrate in the CSF) had a MI below this value (horizontal dotted line). The vertical dotted line separates the samples with a "negative" CSR (<1) from those with a "positive" CSR (>1). A high proportion of CSF samples in the upper right or lower left quadrant indicate a positive association.

As a chemotactic gradient is a major determinant of chemotactic activity, the concentration of each chemokine was also measured in the corresponding serum samples (Fig. 2b, e, and 2h) and the CSF-to-serum ratio (CSR) was calculated. While an increased CSR in LNB was detected for CCL19 (CSR = 5, Fig. 2f) and a marginal difference was found for CXCL12 (CSR = 1.1, Fig. 2i), the most impressive gradient, 28.6 fold higher than that of CCL19, was found for CXCL13 (CSR = 143, Fig. 2c). In a next step, the CSR of the three chemokines was plotted with the MI of the respective CSF samples. As visualized in Fig. 3, the CSR of both, CXCL13 and CCL19, but not CXCL12 was found to be associated with an elevated MI. Combining the high chemotactic gradient and its association with the MI, CXCL13 appears to be the major candidate for a functional role in B cell recruitment to the CSF in LNB.

### Blocking the migration with neutralizing chemokine antibodies

**Figure 4:**
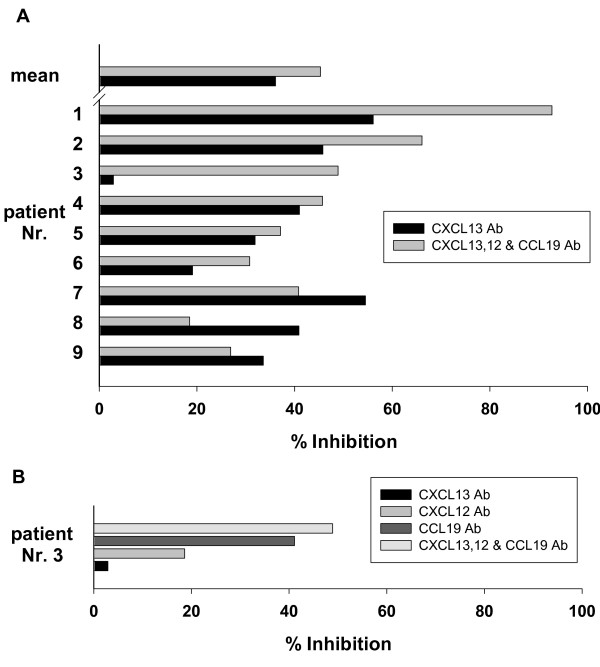
**Inhibition of migration with neutralizing antibodies**. (A) Inhibition of the chemotactic effect of CSF samples from 9 LNB patients (same enumeration as in table 1) with the indicated antibody (in % compared with the respective isotype control). (B) All antibodies were used separately in patient Nr. 3, where the CXCL13 antibody alone had no relevant effect.

The functional role of the B cell attracting chemokines was further analysed using neutralizing antibodies. CSF samples of LNB patients were preincubated with either a neutralizing CXCL13-antibody alone or all three (CCL19, CXCL12 and CXCL13) antibodies together. Compared with the isotype controls, blocking the CXCL13 activity alone reduces the percentage of migrated cells up to 56% (mean 36.2%, p = 0.005) (Fig. 4a). In more than 50% of the samples, the addition of CCL19 and CXCL12 did not further substantially decrease the migratory activity. However, in patient Nr. 3, neutralizing CXCL13 alone had hardly any effect, while all three antibodies together reduced the migration by nearly 50%. Therefore, the CCL19- and CXCL12- antibodies were also separately tested in this sample, and the neutralizing capacity of the CCL19-antibody was more than 2-fold higher than that of the CXCL12 antibody (Fig. 4b). Taken together, the inhibition assays show that CXCL13 seems to play the dominant functional role for B cell migration to LNB CSF, but both CCL19 and CXCL12 might contribute in selected samples.

### Additional, yet unidentified chemotactic factors may also be involved

**Figure 5:**
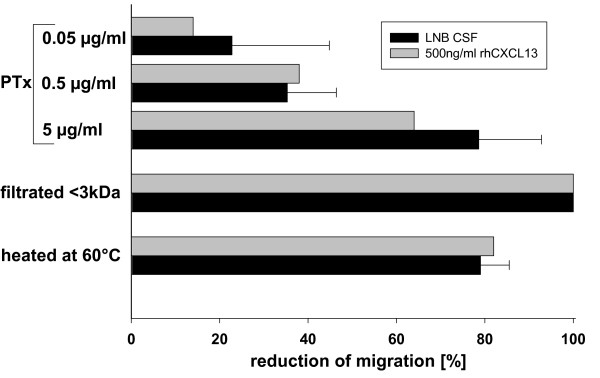
**Inhibition of migration with PTx, heating, or filtration of substances >3 kDa**. CSF samples of LNB patients (n = 3, black bars) or 500 ng/ml rhCXCL13 (mean of two independent experiments, done in duplicates, gray bars) were used as chemotactic agent. Either, the B-cells were preincubated with PTx, or the chemotactic agent was either filtrated to eliminate substances larger than 3 kDa or heated at 60°C prior to the migration assay. The reduction of migration was compared to the untreated B cells, or the untreated chemotactic agents, respectively.

Although our results suggest that CXCL13 is a key determinant of chemotactic activity for B cells in LNB, additional factors appear to be involved as the inhibition has not been complete. To get a first insight into further immune factors involved, we either filtered the CSF samples to eliminate components with more than 3 kDa, denaturized proteins by heating the samples at 60°C or preincubated the B-cells with *Bordetella pertussis *toxin (PTx) to exclude signalling via G_i_-proteins (as used by the chemokines [21]). While PTx treatment and heating reduced the migratory activity by approximately 80%, the filtration of components larger than 3 kDa completely eliminated the chemotactic effect. This pattern was similar, if rhCXCL13 was used as chemotactic agent (Fig. 5). Taken together, these yet unidentified chemotactic factors have to be larger than 3 kDa, are predominantly heat sensitive and signal via G_i_-proteins.

## Discussion

The mechanisms underlying the B cell rich CSF pleocytosis in LNB are still undefined. Here we demonstrate that CSF of LNB patients is chemotactic to peripheral blood human B cells, and that CXCL13 is a major regulator of B cell recruitment in acute LNB.

The cells that migrated towards the CSF of LNB patients in our *in vitro *setting were predominantly CD27^+ ^B cells. The surface marker CD27^+ ^on B cells indicates, that these cells are activated and can produce 5- to 100-fold more immunoglobulins than CD27^- ^cells [22]. Earlier studies on the immune response during the course of *B.b*. meningoradiculitis revealed, that large numbers of B cells in the CSF and a prominent intrathecal production of antigens are characteristic for LNB [13], suggesting that these B-cells are from the (CD27^+^) mature type. The exact rate of CD27^+ ^cells in the CSF in LNB, however, has not been determined so far. Results from patients with other neuroinflammatory disorders like multiple sclerosis, viral infections, or neurosyphilis showed that 85% of the total B cell population in the CSF are CD27^+^, in contrast to 31% in the peripheral blood [23]. Taken these studies together, CD27^+ ^B cells appear to be the main migrating B cell population in neuroinflammation, both *in vivo *and in our *in vitro *experimental setup. Therefore, our *in vitro *model seems to be a suitable tool to search for the responsible chemotactic agent for B cell immigration in LNB.

This chemotactic agent should fulfil four criteria: a) it has to attract B cells, b) it has to be elevated in the CSF of LNB patients and there must be a gradient over the blood-CSF barrier, c) there should be an association between the gradient and the intensity of B-cell-migration, and d) its neutralization should abrogate the observed B cell migration to LNB CSF. The four known major B cell attracting chemokines are CCL19, CCL21, CXCL12, and CXCL13, as shown by Brandes et al. [14]. From recent studies, it is known that CXCL13 is elevated in the CSF, but not the blood of LNB patients [10,16]. In the current study, we further showed that rhCXCL13, when used in concentrations as found in LNB CSF samples, is chemotactic to B cells. Data concerning the concentration of other B-cell attracting chemokines in LNB CSF are rarely found in the literature. Only CXCL12 was explicitly measured in a small collective of 6 LNB patients with a mean of 24.1 ng/ml in the CSF of LNB patients and 5.5 ng/ml in NIND patients [24,25]. Elevated levels of CCL19 in the CSF of patients with various infectious diseases (including LNB patients) were determined by two study groups [26,27] with a mean value between 173 - 250 pg/ml compared to 26 - 62 pg/ml in NIND patients. CCL21 in contrast was not found at measurable concentrations in any of these studies. Exact data for LNB patients or serum concentrations to calculate the gradient of CCL19 or CCL21 were not available. Therefore, we measured the concentrations of CCL19, CCL21, CXCL12 and CXCL13 in CSF/serum pairs of patients with LNB. The most prominent results were found as expected for CXCL13 [10,16] with a more than 300 fold difference in the CSF between LNB and NIND patients and a mean CSR of approximately 150.

All of the LNB patients had not been treated with antibiotics before the lumbar puncture. One of the NS patients received an antibiotic therapy for two weeks before CSF sampling and showed the lowest CXCL13 CSF value of all NS patients. This observation fits perfectly to recent studies demonstrating that (I) viable spirochetes are needed for the production of CXCL13 in monocytic cells [16] and (II) CSF CXCL13 concentrations rapidly decreases under antibiotic therapy in LNB patients [10].

The difference between the patient groups is less impressive for CCL19 but still compatible with a functional chemotactic role: The mean CSF concentration is 8 fold higher than in NIND patients and the mean CSR equals 5.

The results obtained for CXCL12 are lower than those from Pashenkov et al. [24], but the high constitutive expression of CXCL12 in the CNS and the lack of an elevated CSR, arguing against a functional role for B cell immigration, was confirmed in our study. The concentration of CCL21 in the CSF, as expected from previous studies, is hardly measurable in any patient group and a functional role therefore very unlikely. In view of these results, CXCL13, but also CCL19 and less likely CXCL12 were identified as putative chemotactic agents in LNB. This was further underlined by the association analysis. High CXCL13 and CCL19, but not CXCL12 concentrations in LNB CSF were associated with a high MI. Finally, the neutralization experiments underlined the key role of CXCL13, since preincubation with anti-CXCL13 antibodies significantly reduced the migration activity of B cells. Therefore CXCL13 fulfils all criteria as stated above.

CCL19 instead, which signals via the CCR7 receptor, appears to play a functional role for B-cells only in single cases (as seen in patient Nr. 3, Fig. 4). However, this chemokine could be important for the immigration of T-lymphocytes instead, as suggested in the literature. Activated T-lymphocytes strongly upregulate CCR7 and efficiently migrate to CCL19 [28], and T-lymphocytes found in the CSF of patients with (not further specified) inflammatory CNS-diseases are CCR7 positive [27]. As CCL19 is much stronger chemotactic for T than for B cells, its role in LNB could be the attraction of T cells, but this would have to be determined in further studies.

For the association analysis as shown in Figure 3, the cut-off for an increased migration index was set at 80, as the CSF of all NIND-patients had a migration below this value. As seen in Figure 3A and 3B and also in Figure 1A, there are four CSF samples from LNB patient with a migration index below 80. The one of them with the highest MI also has a high CSR for CXCL13 and resembles in its basic CSF findings the other LNB patients (cell count 368/μl, albumin CSF/serum quotient 19.1). The three others, however, appear to be a distinct group. They are combining a low CSR for both CXCL13 (7-18.5) and CCL19 (0.7-1.7) with a low migration index. Compared to the remaining LNB patients, they also have a lower cell count (19 ± 10 vs. 146 ± 152 cells/μl, p < 0.01), albumin CSF/serum quotient (7.2 ± 5.2 vs. 19.6 ± 13, p = 0.02), and in none of them, plasma cells are found in the CSF (in contrast to a mean of 5% in the other LNB patients, p < 0.01.). Although these data should be interpreted with caution, given the small sample size of this subgroup, the observation that in all three patients low-grade inflammatory CSF changes were paralleled by very low CSRs for CXCL13 and CCL19 (as compared to other LNB patients) further underlines the functional role of both chemokines. It also has to be mentioned that the less pronounced CSF alterations in this LNB subgroup are not reflected by less (or different) clinical symptoms or by clear differences in age or gender. However, we are not aware of the duration between the onset of symptoms and lumbar puncture. Both the quantity and quality of CSF abnormalities (e.g. CSF pleocytosis and CSF chemokine levels) may change over time after infection, which may be an interesting topic to be addressed in future studies.

As the inhibition of CXCL13, CCL19 and CXCL12 does not completely abrogate the chemotactic activity of LNB CSF samples, additional factors have to be involved. In a study by Dubois et al., the supernatant of cultured dendritic cells was found to be chemotactic for B cells [29]. As the supernatant was insensitive to PTx treatment, the authors stated that the relevant factors have to be different from classical chemoattractants. In our study, however, the chemotactic activity of both, rhCXCL13 and LNB CSF was reduced by around 80% by interfering with the PTx pathway, and also heat treatment. In addition, the chemotactic activity of LNB CSF samples and rhCXCL13 was completely blocked by eliminating all substances larger than 3 kDa. Chemokines weigh between 6 and 14 kDa [30], signal predominantly via the PTx-sensitive pathway [31], and are as proteins heat sensitive. This indicates that, besides CXCL13, another "similar" chemokine could be responsible for the remaining chemotactic activity after blockade of CXCL13. Another possible candidate could be the complement, as the size of complement factors ranges from 24 to 410 kDa, they are heat sensitive and - as shown for C5a - signal via PTx-sensitive G proteins [32]. In addition, it has been demonstrated, that *B.b*. can induce the production of complement in vitro and in vivo [33,34] and that B cells respond chemotactic to C5a [35]. However, there are even more substances to be considered: components of the Borrelia themselves could also contribute to the chemotaxis. In a study by Benach et al., large proteins of the borrelial membrane like the Outer surface protein A or flagellin had a chemotactic effect on leucocytes [36]. Taken together, further studies are warranted to identify all factors involved in B cell attraction.

## Conclusions

In summary, CXCL13 plays a key role for the immigration of B cells into the CSF in LNB. Therefore - apart from its unquestioned role in dictating homing and motility of lymphocytes in lymphoid tissues [37] - this potent B cell attracting chemokine appears to be also important for attracting B cells to sites of acute bacterial infection.

## Competing interests

The authors declare that they have no competing interests.

## Authors' contributions

TAR and UK have conceived of the study, TAR has established the experimental setting and written the manuscript, AP has carried out most of the experiments, MA has done the FACS analysis, MW and SK have collected the CSF and serum samples, CS and MK have participated in the design of the study and the statistical analysis and helped drafting the manuscript. HWP and UK have participated in the design and coordination of the study and helped to draft the manuscript. All authors read and approved the final manuscript.
